# Rapamycin Inhibits Senescence and Improves Immunomodulatory Function of Mesenchymal Stem Cells Through IL-8 and TGF-β Signaling

**DOI:** 10.1007/s12015-024-10682-x

**Published:** 2024-02-10

**Authors:** Aaron J Sheppard, Kristin Delgado, Ann Marie Barfield, Qinqin Xu, Patrick A Massey, Yufeng Dong, Richard S Barton

**Affiliations:** 1 School of Medicine, LSU Health Shreveport, Shreveport, LA USA; 2 Department of Orthopedic Surgery, LSU Health Shreveport, Shreveport, LA USA

**Keywords:** Mesenchymal stromal cells, Rapamycin, Senescence, Cell sheet culture

## Abstract

Mesenchymal stromal cells (MSCs) grown in high-density monolayers (sheets) are promising vehicles for numerous bioengineering applications. When MSC sheets are maintained in prolonged cultures, they undergo rapid senescence, limiting their downstream efficacy. Although rapamycin is a potential agent that can inhibit senescence in cell cultures, no study has investigated rapamycin’s effect on MSCs grown in high-density culture and its effect on downstream target gene expression. In this study, placental-derived MSCs (PMSCs) were seeded at high density to generate PMSC sheets in 24 hours and were then treated with rapamycin or vehicle for up to 7 days. Autophagy activity, cell senescence and apoptosis, cell size and granularity, and senescence-associated cytokines (IL-6 and IL-8) were analyzed. Differential response in gene expression were assessed via microarray analysis. Rapamycin significantly increased PMSC sheet autophagy activity, inhibited cellular senescence, decreased cell size and granularity at all timepoints. Rapamycin also significantly decreased the number of cells in late apoptosis at day 7 of sheet culture, as well as caspase 3/7 activity at all timepoints. Notably, while rapamycin decreased IL-6 secretion, increased IL-8 levels were observed at all timepoints. Microarray analysis further confirmed the upregulation of IL-8 transcription, as well as provided a list of 396 genes with 2-fold differential expression, where transforming growth factor-β (TGF-β) signaling were identified as important upregulated pathways. Rapamycin both decreased senescence and has an immunomodulatory action of PMSCs grown in sheet culture, which will likely improve the chemotaxis of pro-healing cells to sites of tissue repair in future bioengineering applications.

**Graphical bstract:**

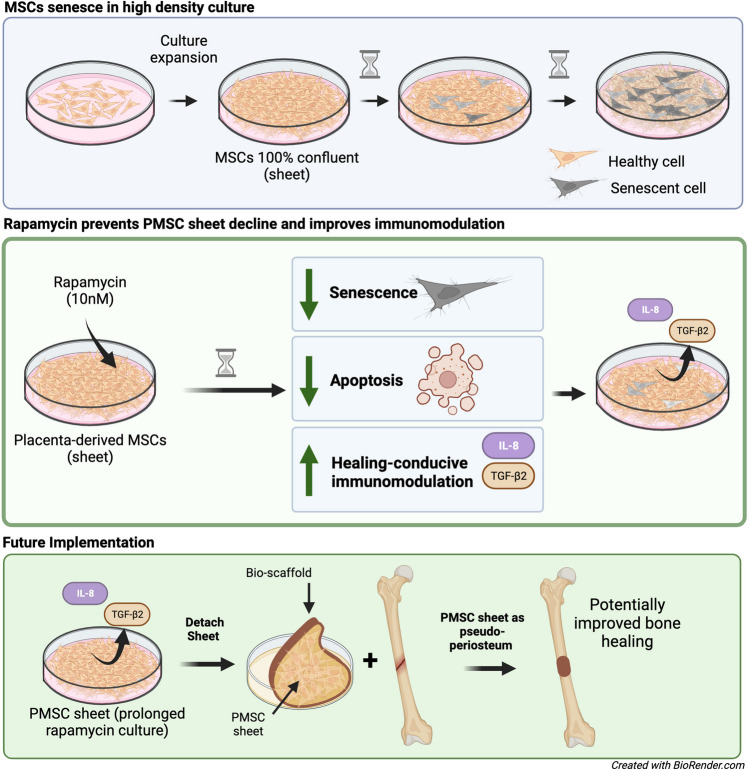

**Supplementary Information:**

The online version contains supplementary material available at 10.1007/s12015-024-10682-x.

## Introduction

Mesenchymal stromal cells (MSCs) grown in high-density monolayers (MSC sheets) serve as promising therapeutics for a variety of tissue regeneration applications [1, 2]. Due to their multipotent nature, MSCs have been in the spotlight as promising treatment vehicles for decades, including to augment bone repair, improve cartilage healing, and modulate the immune response [3, 4]. When grown in sheets, MSCs secrete extracellular matrix components and have cell-to-cell interactions that are conducive for a healing response, as well as contribute to the sheets abilities to survive in host tissues and their successful transplantation [5–7]. These MSC sheets can be easily transferred from culture dishes and utilized for numerous regenerative medicine and tissue engineering applications, including bone defect repair [6]. For such bioengineering applications, placenta-derived MSCs (PMSCs) are a popular cell line due to their abundance, ease of isolation, and potentially superior healing properties, including in bone defect repair [8–10].

Despite the great promise of MSC sheets, one major challenge is that they undergo rapid senescence in as early as 3 days in high-confluency culture [11, 12]. There is a critical need for techniques to prolong the function of these MSCs and optimize their treatment capacity. MSCs within high-confluency culture acquire the hallmarks of aging, such as the absence of proliferative markers, increased senescence-associated β-galactosidase (SA β-GAL) activity, expression of the senescence-associated secretory phenotype (SASP), and expression of cell cycle inhibitors [11]. For many therapeutic applications, especially with transplantations for bone regeneration applications, these MSC sheets must be grown in culture for several days, or weeks, before transplantation. Several examples of the need to prolong culture of MSC sheets is (1) to allow time for an ECM to be established, (2) to pre-treat with agents to prime the sheets for the desired therapeutic application, and (3) to grow multiple-layer MSC sheets [13–17]. For these approaches to be possible, there is a great need for techniques, or culture-enriched conditions, to prolong MSC function.

Rapamycin, the natural mechanistic target of rapamycin (mTOR) inhibitor, is a promising remedy to potentially prevent senescence and improve the function of MSC sheets. While it is currently FDA-approved as a cancer treatment and transplant rejection prevention agent, rapamycin has arguably received the most press for its anti-aging and lifespan prolonging effects in mice and other model organisms [18–20]. The mTORC1 complex is poised to integrate the status of growth factors, amino acids, and cellular energy reserves to coordinate a broad range of cellular processes, including proliferation, survival, protein synthesis, mitochondrial biogenesis, adipogenesis, lipid metabolism, and more [19]. Many have shown that rapamycin decelerates cellular senescence in various cell types and model organisms by up-regulating autophagy as well as several novel pathways [19, 21–23]. While inhibiting mTOR signaling has proven to decelerate senescence by influencing hallmark, or global, cellular processes [24, 25], less work has been done to pinpoint specific pathways (downstream from mTOR) that may drive these cellular process outside of the oncology literature. Further, it is likely that these downstream molecular changes (1) depend on the cell type/cell origin and (2) may affect important pathways which are crucial to the desired bioengineering application such as for bone repair.

To our knowledge, rapamycin’s senescence-halting properties have not been tested in PMSCs, or in MSCs, grown in prolonged sheet culture. Thus, the primary aim of this preliminary study was to test if rapamycin could prevent senescence and functional decline of PMSCs grown in sheet culture to preserve them for downstream applications. The second aim was to explore the molecular pathways affected by rapamycin, with an interest on genes not only involved in aging, but in the tissue healing response.

## Materials and Methods

### Stem Cell Isolation and Culture

Placentas delivered during routine birth were immediately collected. Since the placenta is considered a medical waste, no consent from the patients was needed. Collection of human placentas for MSC isolation was approved by the IRB at Louisiana State University Health Shreveport (LSUHS), and MSC isolation was processed at the Department of Gynecology and Obstetrics, LSUHSC-S. The procedures for PMSC isolation, culture, and characterization were performed as described before [10]. For these experiments, passage 6–8 PMSCs were cultured in 6-well plates at a specific density to rapidly induce PMSC sheets after 24 hours. After 24 hours, the cells were washed and treated with either rapamycin (10nM) or equal volume vehicle (DMSO). Treatment media was changed every 2 days, unless specified. To confirm efficacy of the chosen rapamycin dose, PMSC sheets were stained for autophagic vesicles (Abcam, ab139484) after 4 days of rapamycin treatment. The cells were images and then analyzed via ImageJ software (National Institutes of Health, Bethesda, MD) [26]. All following experiments were repeated in triplicate.

### Functional Assays

PMSC sheets were treated with rapamycin or vehicle for 4 and 7 days. PMSC sheets were stained for Senescence associated β-Galactosidase activity (SA β-GAL) at both timepoints (Cell Signaling, #9860). Sheets were imaged on an EVOS phase-contrast microscope (Advanced Microscopy Group, Bothell, WA) at 40X, and quantified (percent-positive (%-positive) area) using ImageJ. Cell size and granularity between groups were indirectly assessed via forward and size scatter, respectively, via flow cytometry. Apoptosis was detected using Annexin V FITC apoptosis detection kit (BD Biosciences, San Jose, CA). Flow cytometry was used to analyze the cells and determine the percentage of cells in different apoptosis stages. As a secondary measure of apoptosis, a Caspase-Glo 3/7 luminescence assay (Promega, #G8090) was used to assess caspase 3 and 7 activity at day 4 and 7 of sheet culture. The secretion of senescence-associated cytokines IL-6 and IL8 were measured in the sheet culture media at 24, 48, and 96 hours after treatment using enzyme-linked immunosorbent assays (ELISA) (IL-6 assay: Invitrogen, #EH2IL6; IL-8 assay: R&D Systems, #D8000C).

### Microarray and Network Analysis

Total RNA was extracted from PMSC sheets treated for 24 hours. This experiment was repeated on three independent occasions. RNA quality was determined with Agilent TapeStation RNA assay (Agilent Technologies). RNA quantity was assessed with the Qbit Broad Range RNA assay (Invitrogen). RNA samples were labeled for hybridization according to the standard GeneChip WT PLUS Reagent Kit. Approximately 150 nanograms of total RNA were input into this assay. The fragmented, biotin-labeled sense-strand ss-cDNA were hybridized to Affymetrix GeneChip Clariom S Human Arrays. Pixel intensity measurement, feature extraction, data summarization, normalization and differential gene analysis were performed in Transcriptome Analysis Console (TAC) version 4.0. Arrays were normalized using the SST-RMA (Signal Space Transformation Robust Multi-Chip Analysis) algorithm, which consists of background adjustment, quantile normalization and summarization. The integrated WikiPathways tool within TAC was used to explore enriched pathways and genes by rapamycin treatment. Briefly, the TAC tool uses a Fisher’s Exact Test to assess whether the observed overlap between the gene set of interest and the pathway is statistically significant compared to what would be expected by chance. The p-value obtained after converted to -log10 is called the Significance value. Further, individual genes with a significant 2-fold difference in gene expression (and false discovery rate-adjusted p-value < 0.05) were selected for input into the search tool for retrieval of interacting genes (STRING) (https://string-db.org) database using the geneset-based analysis to acquire protein-protein interactions (PPIs). The interaction sources consisted of STRING’s text mining, database, experiments, co-occurrence, and co-expression with an interaction score of greater than 0.7 (high confidence) to construct the PPI network.

### Statistics

All statistical analyses were performed using GraphPad Prism version 10.0.0 for MacBook (GraphPad Software, Boston, Massachusetts USA). Data are presented as mean ± standard deviation (SD). A p-value less than 0.05 was used as the threshold for significance. For multiple tests carried out on one sample, a Bonferroni correction was applied.

## Results and Discussion

To test rapamycin’s ability to improve the functionality of PMSCs in long term sheet culture, a set of functional experiments were carried out to assess the global effects of rapamycin. In a preliminary experiment, we found that PMSC cells had little-to-no colony forming unit (CFU) potential after being cultured for as little as 4 days in high-density sheet culture. However, we found that rapamycin (10 nM) treatment could retain CFU potential of PMSCs grown in this way (Supplemental Fig. S1A). Further, we also found that PMSCs grown in high-density sheet culture were difficult to differentiate into osteoblasts, where cells began to shown signs of cell detachment and apoptosis after 7–14 days of osteogenic induction, as shown and explained in Supplemental Fig. S1B. However, PMSC sheets pre-treated with rapamycin (10 nM) had far less cell detachment and could maintain culture for 3–4 weeks. While preliminary experiments found that rapamycin decreased the osteogenic potential of PMSC sheets, it was evident that they had improved attachment and improved ability to withstand the stressful high-density culture and highly metabolic process of osteogenesis (Supplemental Fig. S1B). These finding led our group to further investigate rapamycin’s effect on more defined functional parameters, and then to investigate the molecular underpinnings of its ability to improve the function of PMSC sheets. First, we assessed autophagy, senescence, apoptosis, and senescence-associated cytokine secretion.

### Rapamycin Increases Autophagy and Prevents Senescence of High-Density PMSC Monolayers

**Fig. 1:**
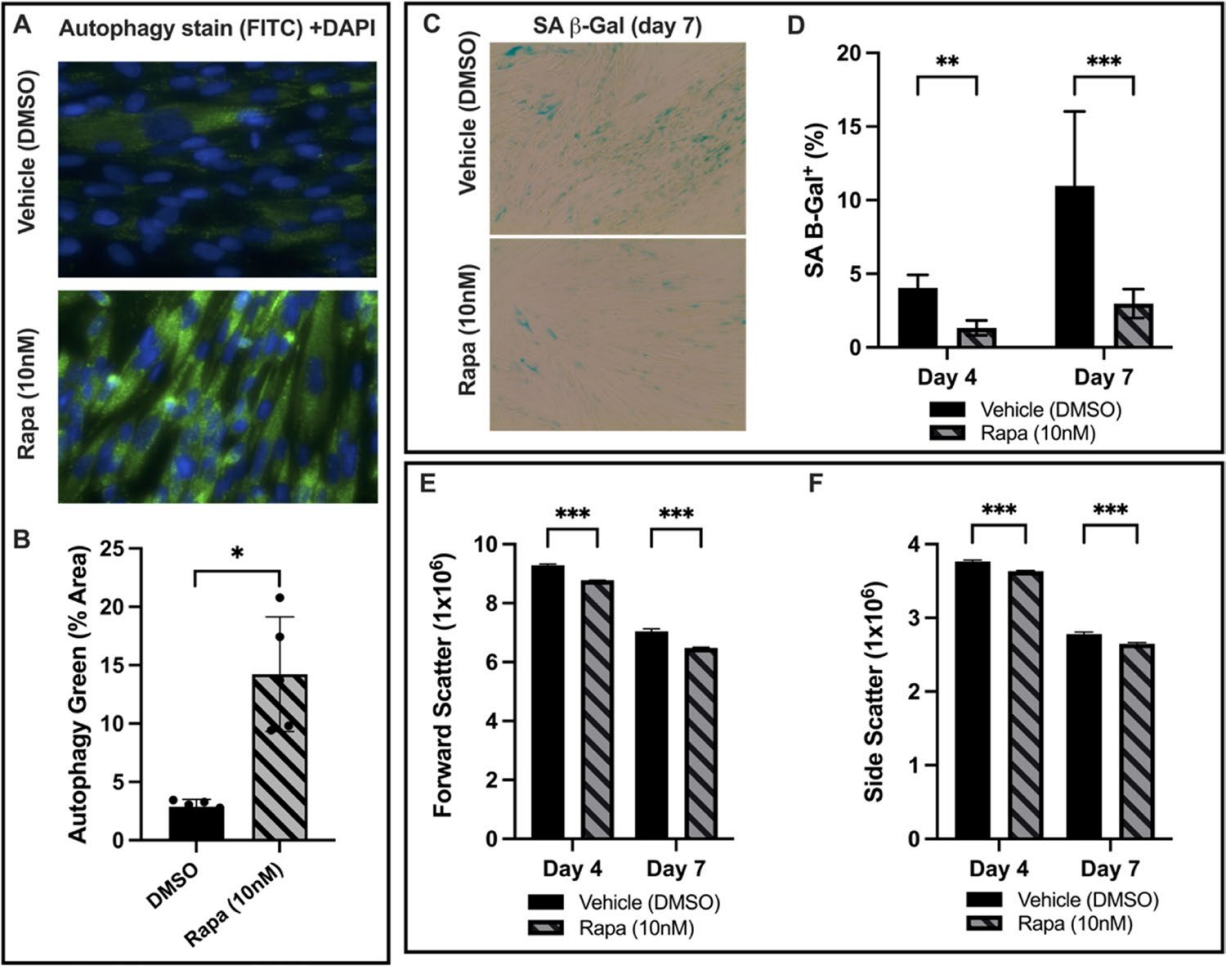
**A** PMSCs in high-density monolayer were stained for autophagy-associated vacuoles and DAPI. The %-positive area of autophagy stain was normalized to DAPI to quantify the relative change of autophagy level. At day 4, the PMSCs with 10nM rapamycin showed a marked increase in autophagy. **B** The rapamycin treated cells had a 3.94-fold increase in autophagy-associated stain compared to the vehicle control (*p* = 0.004, *n* = 5). **C** High-density PMSC monolayers were stained for senescence-associated β-galactosidase activity on day 4 and 7. **D** At day 4, the rapamycin group had 67.7% less SA β-Gal positive area (*p* = 0.0067, *n* = 15). At day 7, the rapamycin group had 72.9% less SA β-Gal positive area (*p* = 2.16x10^−11^, *n* = 15). **E** Forward scatter and (**F**) side scatter were used as measures of relative cell size and complexity. The rapamycin group had a 5.52% and 8% decrease in forward scatter at day 4 and 7, respectively. Additionally, side scatter decreased by 3.4% and 4.7% at day 4 and 7, respectively. Note: *p < 0.05, **p < 0.01, ***p < 0.0001

As shown in Fig. 1A, autophagy-associated vacuoles were stained and imaged at day 4 of rapamycin treatment. The relative autophagy level was quantified by determining the percentage of area that stained positive for autophagy-associated green (FITC) stain, normalized to nuclear stain (DAPI). Rapamycin at 10 nM markedly increased the level of autophagy-associated vacuoles. For the vehicle group, the mean percent-positive autophagy stain was 2.88 ± 0.64%, compared to 14.23 ± 4.9% for the rapamycin group (Fig. 1B), which is a 3.94-fold increase in autophagy-associated stain (*p* = 0.004, *n* = 5). Senescence of high-density PMSC monolayers was assessed by quantifying %-positive area of SA β-Gal stain. At both timepoints, the rapamycin group showed less SA β-Gal activity (Fig. 1C). At day 4, the mean %-positive area of SA β-Gal stain for the vehicle group was 4.02 ± 0.9%, compared to 1.3 ± 0.5% for the rapamycin group (p = 0.007, *n* = 15). At day 7, the mean %-positive area for the vehicle was 10.96 ± 5.05%, compared to 2.97 ± 0.99% for the rapamycin group (Fig. 1D). Thus, rapamycin accounted for an average 67.7% decrease (*p* = 0.0067, *n* = 15) and 72.9% (*p* = 2.2x10^−11^, *n* = 15) decrease in SA β-Gal activity at day 4 and 7, respectively. As shown in Fig. 1E and F, PMSCs treated with rapamycin also had decreased forward and side scatter (size and granularity, respectively), when analyzed with flow cytometry. Rapamycin-treated PMSC sheets had a 5.52% decrease in forward scatter (*p* = 1.4x10^−6^, *n* = 3) at day 4 and 8.0% (*p* = 6.8x10^−7^, *n* = 3) at day 7. Similarly, rapamycin decreased side scatter by 3.4% (*p* = 4.6x10^−4^, *n* = 3) at day 4 and 4.7% (*p* = 4.1x10^−5^, *n* = 3) at day 7.

### Rapamycin Decreases Apoptosis of High-Density PMSC Monolayers

**Fig. 2:**
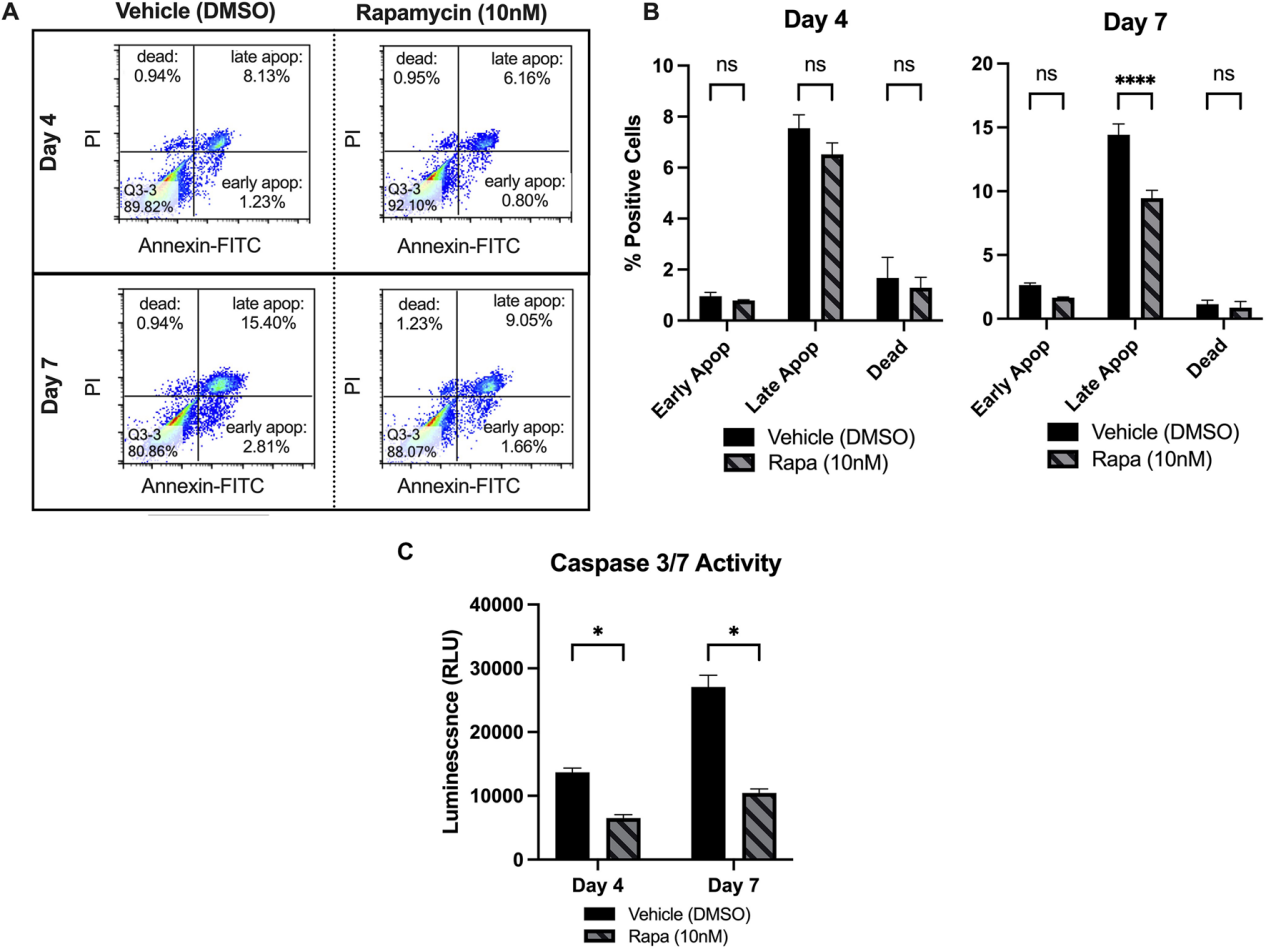
**A** Annexin-V and Propidium Iodide (PI) staining was used to assess apoptosis at days 4 and 7 of sheet culture, where the bottom right segment of each plot represents the population of cells in early apoptosis, the top right segments represent the late apoptosis populations, and the top left segments represent the dead cell populations (**B**) At day 4, the rapamycin treated PMSCs had less cells in early and late apoptosis, however, these decreases did not reach statistical significance. The rapamycin group had an average of 0.79 ± 0.026% of cells in early apoptosis, compared to 0.95 ± 0.157% for the vehicle group (*p* = 0.69, *n* = 3). Further, the rapamycin group had 6.5 ± 0.46% of cells in late apoptosis, compared to 7.54 ± 0.53% for the vehicle group (*p* = 0.02, *n* = 3). The rapamycin PMSCs also had a slight, non-significant decrease in the percentage of dead cells (*p* = 0.33, *n* = 3). At day 7, the rapamycin group had a significant decrease in the percentage of cells in late apoptosis (9.46 ± 0.62% of rapamycin-treated PMSCs vs. 14.42 ± 0.85% for control, *p* = 3.8x10^−8^, *n* = 3). Further, the rapamycin-treated PMSCs had an insignificant decrease in number of cells in early apoptosis (1.66 ± 0.05% versus 2.63 ± 0.175, *p* = 0.033, *n* = 3). The rapamycin-treated PMSCs had a nonsignificant decrease in the percentage of dead cells (0.866 ± 0.5% vs. 1.14 ± 0.31%, *p* = 0.51, *n* = 3). **C** Caspase 3 and 7 activity was assessed as a secondary measure of apoptosis at day 4 and 7, which confirmed a decrease in apoptosis at both timepoints. The rapamycin treatment group had a 52.4% decrease at day 4 (*p* = 1.3x10^−4^, *n* = 3) and a 61.4% decrease at day 7 (*p* = 1.3x10^−4^, *n* = 3). Note: *p < 0.05, **p < 0.01, ***p < 0.0001

The percentage of cells in early and late apoptosis, as well as the percentage of dead cells, was assessed at day 4 and 7. Figure 2A displays an example of flow cytometry result from one replicate, where the rapamycin-treated PMSCs had a decrease in early and late apoptosis populations at each timepoint. At day 7, the rapamycin group had a significant decrease in the percentage of cells in late apoptosis (9.46 ± 0.62% compared to 14.42 ± 0.85%, *p* = 3.8x10^−8^, *n* = 3) (Fig. 2B). Further, the rapamycin-treated PMSCs had an insignificant decrease in number of cells in early apoptosis (1.66 ± 0.05% compared to 2.63 ± 0.175, *p* = 0.033, *n* = 3) and a nonsignificant decrease in dead cells (0.866 ± 0.5% compared to 1.14 ± 0.31%, *p* = 0.51, *n* = 3). At day 4, the rapamycin treated PMSCs had less cells in early and late apoptosis, as well as less dead cells. However, these decreases did not reach statistical significance. To further confirm rapamycin decreases apoptosis, relative caspase 3/7 activity was assessed after 4 and 7 days in high-density culture (Fig. 2C). The caspase 3/7 assay measures luminescence produced by caspase 3/7 activity, allowing the comparison of relative caspase activity. At day 4 and 7, the rapamycin treatment group had a 52.4% decrease (*p* = 1.3x10^−4^, *n* = 3) and a 61.4% decrease (*p* = 1.3x10^−4^, *n* = 3) at day 4 and 7, respectively. This finding of rapamycin’s ability to reduce apoptosis of PMSCs in sheet culture helps partially explain the improved CFU potential (Supplemental Fig. S1A), as there are more viable cells in the rapamycin group with the ability to resume proliferation.

### Senescence-Associated IL-6 and IL-8 Secretion

**Fig. 3:**
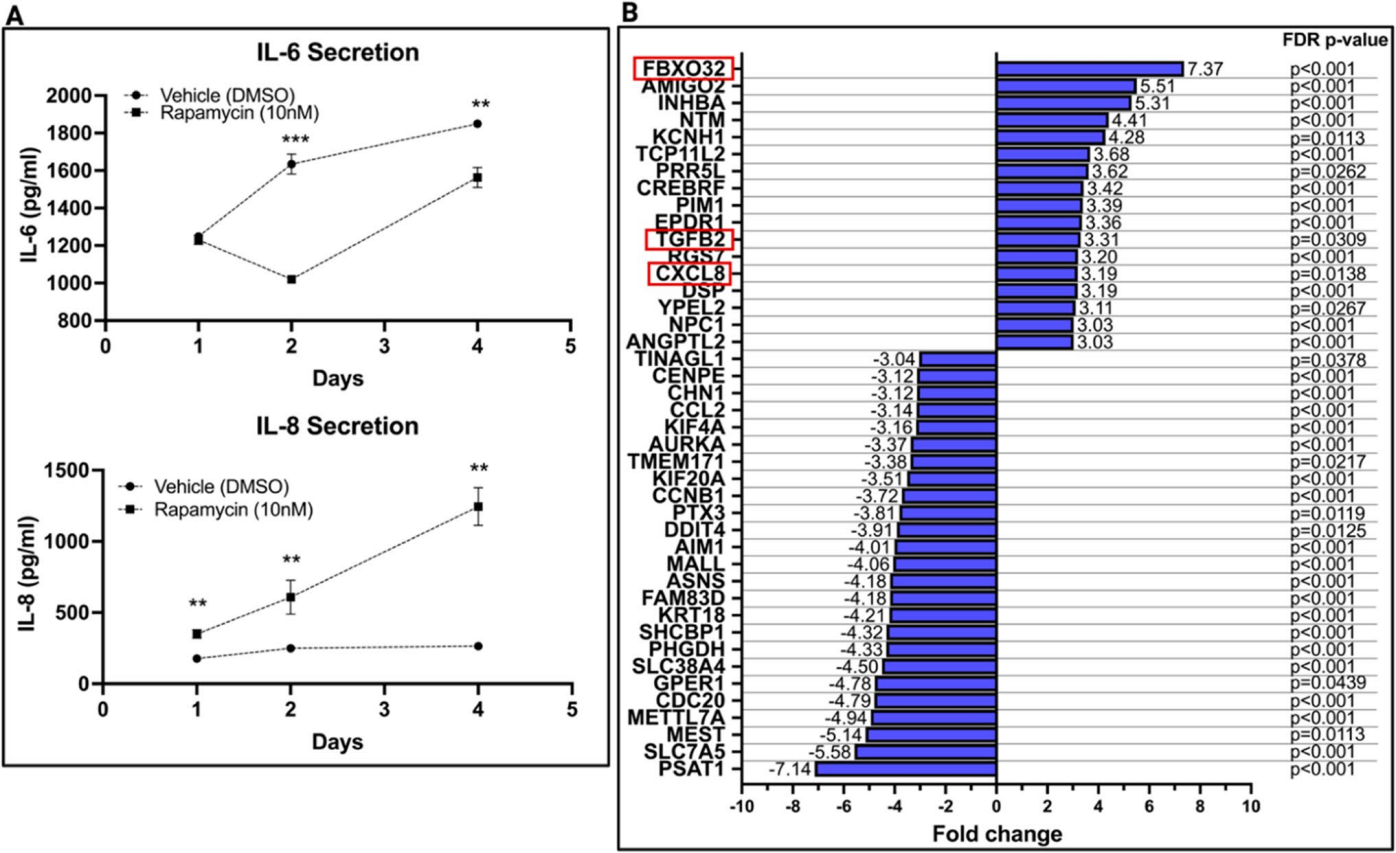
**A** The concentration of IL-6 and IL-8 secreted in culture media was assessed via ELISA at 1, 2, and 4 days of sheet culture. At day 1, there was an insignificant difference in IL-6 concentration between groups (*p* = 0.32). At day 2, the rapamycin group had 37.6% less IL-6 secretion (1020 ± 18.7 pg/ml compared to 1634 ± 53.0 pg/ml of the DMSO group, *p* < 0.001, *n *= 3). At day 4, the rapamycin group had 15.5% less IL-6 secretion (1563 ± 53.5 pg/ml compared to 1850 ± 15.1 pg/ml of the DMSO group, *p* < 0.001, *n *= 3). Further, the rapamycin-treated group had a 1.98-, 2.44-, and 3.7-fold greater IL-8 secretion at day 1, 2, and 4 respectively (*p* < 0.001, *p* < 0.01, and *p* < 0.01, respectively). Data represents mean ± SD for *n* = 3 experiments. Note: *p < 0.05, **p < 0.01, and ***p < 0.0001. **B** A microarray was performed to assess differential gene expression profiles for rapamycin-treated PMSC sheets. The gene expression fold change (relative to DMSO-treated PMSC sheets) for 42 genes with at least a 3-fold change and FDR-adjusted p-value of < 0.05 is presented. Three genes of particular interest (FBXO32, CXCL8, and TGFB2) are highlighted by red boxes. All gene abbreviations and full list of gene expression data (with at least 2-fold change in gene expression) are presented in Supplemental Table 1

Rapamycin-treated PMSC sheets had decreased section of IL-6 at 48 and 96 hours of treatment compared to the control group (Fig. 3A). Rapamycin-treated PMSC sheets showed a decrease in IL-6 secretion from day 1 (1231 ± 26 pg/ml, *n *= 3) to day 2 (1020 ± 18.7 pg/ml, *n *= 3). The IL-6 secretion at day 2 was significantly less than the control group (1020 ± 18.7 pg/ml compared to 1634 ± 53.0 pg/ml of the DMSO group, *p* = 4.6x10^−5^, *n* = 3). From day 2 to 4, the rapamycin-treated cells began to increase secretion of IL-6 from baseline, however, this secretion was still significantly less than the control group (1563 ± 53.5 pg/ml compared to 1850 ± 15.1 pg/ml of the DMSO group, *p* = 8.7x10^−4^, *n* = 3). The difference in IL-6 secretion between the treatment and control group was insignificant after 24 hours of treatment, with an average difference of only 17.83 pg/ml (*p* = 0.321, *n* = 3). IL-8 levels, however, were significantly increased in the rapamycin group compared to DMSO at all three time points measured (Fig. 3A). After 24 hours, rapamycin increased IL-8 secretion by 1.98-fold (349.8 ± 30.6 pg/ml compared to 176.7 ± 3.1 pg/ml for the DMSO group, *p* = 6.2x10^−4^, *n* = 3). At 48 hours, rapamycin increased IL-8 secretion by 2.44-fold (608.6 ± 119.4 pg/ml compared to 249.3 ± 24.0 pg/ml, *p* = 0.0069, *n* = 3). Rapamycin continued to increase IL-8 secretion at 96 hours, where the treatment group had 3.7-fold higher levels of IL-8 (1245 ± 132.5 pg/ml compared to 264.8 ± 10.0 pg/ml, *p* = 2.1x10^−4^, *n* = 3).

### Deeper Dive with Microarray and Pathway Analysis

As evident by functional studies, rapamycin has great potential in maintaining PMSCs in high density culture by increasing autophagy and decreasing both senescence and apoptosis. However, the differential regulation of IL-6 and IL-8 was seemingly paradoxical through a senescence lens, and others have shown rapamycin decreases both Il-6 and IL-8 [21]. Given that mTOR is a major regulator of a myriad of cellular processes, it is not surprising that its inhibition could result in complex molecular adaptations, which are likely affected by cell type, culture conditions, and more. To gain a better understanding of the molecular adaptations of PMSC sheets treated with rapamycin, we performed a microarray to assess gene expression alterations after 24 hours of rapamycin (10nM) treatment. A total of 396 genes were differentially expressed (2-fold differential expression and *p* < 0.05) by the rapamycin group. The genes with at least 3-fold difference in expression and an FDR-adjusted p-value of less than 0.05 are presented in Fig. 3B. A full list of all 396 genes (with 2-fold differential expression), along with their unabbreviated names and fold-changes are provided by Supplemental Table 1. Of genes with at least 3-fold change, three were of particular interest: F-box protein 32 (FBXO32), transforming growth factor beta 2 (TGFB2), and chemokine (C-X-C motif) ligand 8 (CXCL8). FBXO32 had the greatest fold-change expression of + 7.37 (FDR p-value = 0.0025). While FBXO32’s actions are not fully characterized, it is a known ubiquitin E3 ligase and targets proteins for degradation that are involved in skeletal muscle integrity, playing a positive role in muscle atrophy. In addition, it has also been shown to have a tumor suppressor role, downregulated in several cancers including ovarian cancer cell lines and esophageal squamous cell carcinoma [27, 28]. Thus, the significant induction of FBXO32 by rapamycin likely plays a central role in halting PMSC proliferation and senescence, as well as decreasing cell size. Further, the upregulation of TGFB2 (+3.31 fold-change, FDR p-value = 0.0309) was an unexpected finding. TGF-β2 signaling is a known to favor bone formation by enriching osteoprogenitors, serving as a chemoattractant for osteoblast-like cells [29, 30]. Lastly, not only does the upregulation of CXCL8, or IL-8, gene expression (3.19-fold upregulation, FDR p-value = 0.0138) confirm our previous ELISA results, but it also suggests that rapamycin has an immunomodulatory effect on PMSCS, as IL-8 is also a well characterized chemoattractant of immune cells and bone healing cells in the context of bone repair applications [31, 32].

**Fig. 4:**
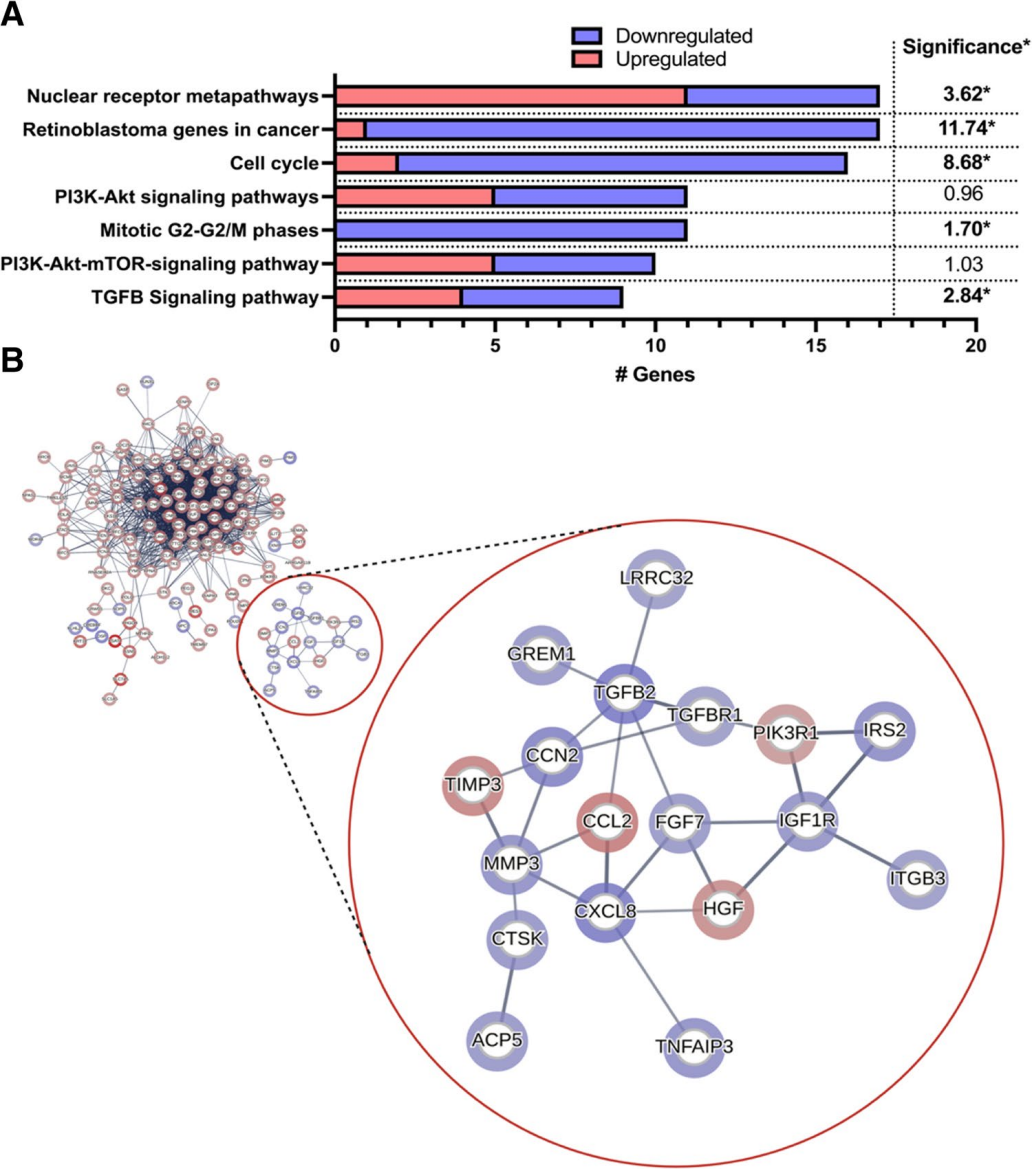
**A** A functional gene enrichment analysis reveals that rapamycin treatment elicits significant differential gene expression within specific pathways (using WIkiPathways tool in Transcriptome Analysis Console) involved in cancer, cell cycle, and PI3K-Akt-mTOR signaling. Of importance, the TGF-β signaling pathway was significantly enriched. The number of genes enriched within each pathway is the x-axis, where blue are downregulated genes, and red are upregulated genes. On the right-most side, the significance (-log10(p-value)) is presented for each enriched pathway. * denotes significance (*p* < 0.05). **B** A total of 316 genes with 2-fold differential expression and FDR-adjusted p-value of less than 0.05 were input into STRING to acquire protein-protein interactions (PPIs). A gene enrichment map was constructed from genes with a 2-fold or greater change in expression between rapamycin and DMSO groups using the STRING database. Panel B focused on a group of 18 protein-protein interactions that are unconnected to the larger group of interactions, which involves cytokine (IL-8), TGF-β2, and PI3K-Akt signaling. Nodes represent proteins encoded by their labelled gene locus. Edges represent protein-protein associations of high confidence (0.7). Halo color and intensity signifies gene down-regulation (red) or up-regulation (blue) and fold-change value, respectively. The nodes correspond to the gene/proteins and the edges represent the interactions. After network analysis and removing unconnected nodes, 310 nodes were identified

Together, the decrease in cell senescence and potentially bone-positive immunomodulatory effects are promising findings for the use of rapamycin on PMSC sheets to augment bone healing applications. To investigate which pathways and signaling cascades are altered by rapamycin to produce these effects, we performed a gene enrichment and protein-protein interaction analysis. The WikiPathway tool within TAC was used to explore significantly enriched pathways (Fig. 4A). For analysis, 316 genes were chosen which had a 2-fold differential expression and an FDR-adjusted p-value less than 0.05 (genes taken from original Supplemental Table 1) and input into STRING’s normal geneset analysis tool. The functional gene enrichment analysis reveals that rapamycin treatment elicits significant differential gene expression within specific pathways involved in cell cycle and proliferation (Fig. 4A). The “retinoblastoma genes in cancer” (significance = 11.74, *p* = 1.82x10^−12^) and “cell cycle” (significance = 8.68, *p* = 2.09x10^−9^) pathways were the most significantly enriched. Of importance, genes within these pathways, including the “mitotic G2-G2/M phases” pathway (significance = 1.70, *p* = 0.020), were predominantly downregulated. 16 of the 17 genes within the “retinoblastoma genes in cancer”, 14 of the 16 genes within the “cell cycle” pathway, and all 11 genes within the “mitotic G2-G2/M phases” pathway were downregulated (Fig. 4A). This halt in proliferation and cancer-associated gene, as well as differential expression of genes within the PI3K-Akt-mTOR pathways is expected with mTOR inhibition and confirms findings from ours, and others, functional assays showing halted cellular replication and metabolism [21, 22]. Consistent with this, MKI67 (Ki-67) and CDC20, which are two widely-accepted markers of proliferation, were significantly downregulated (2.45-fold decrease and 4.79-fold decrease, respectively)(FDR p-value = 0.004 and 0.001, respectively). However, we found the TGF-β signaling pathway to also be significantly enriched (significance = 2.84, *p* = 0.00145).

The PPI map created from STRING is presented in Fig. 4B, which is focused on a group of 18 genes, unconnected to the larger groups of gene interactions. In Fig. 4B, the nodes represent genes encoding their respective protein, while edges resemble the confidence of the interaction (> 0.7), where the band width reflects the strength of data support. The halo color and intensity around each node signifies gene down-regulation (red) or up-regulation (blue). This focused interaction group (red circle in Fig. 4B) highlights the potential key differentially expressed genes within the PI3K-Akt and TGF-β pathways. The interaction network within this cluster was separate from the larger interaction network (full network in Supplemental Fig. 2). As evidenced by the enrichment analysis, the full network consisted primarily of genes involved in the cell cycle, and, broadly, cellular metabolism, ribosomal biogenesis, purine metabolism, cytoskeletal regulation, and protein binding (data not shown), which are likely downstream of mTOR inhibition. However, this focused interaction network highlights a separate cluster of genes, which included CXCL8, TGFB2, and insulin like growth factor 1 receptor (IGF1R).

Rapamycin induced a 2.39-fold upregulation of IGF1R (FDR p-value = 0.012), as well as a 2.61-fold up-regulation of the insulin receptor substrate 2 (IRS2) (FDR p-value = 0.0254). mTOR signaling has been shown to decrease IGF-1/Akt signaling via negative feedback from activated ribosomal protein S6 kinase (S6K) [33, 34]. Thus this finding suggests an increase in IGF-1/Akt signaling due to the relief of negative inhibition, which has been described in other cell lines [33, 35]. Further, rapamycin treatment not only induced a 3.31-fold up-regulation (FDR p-value = 0.031) of TGFB2, but also induced a 2.02-fold upregulation (FDR p-value = 0.0124) of transforming growth factor beta receptor 1 (TGFβR1). Others have also shown that mTOR inhibition activates TGF-β signaling and may play a role in pulmonary fibrosis [36–39]. Osman et al. found that rapamycin increased binding of co-smad proteins to the Smad-binding element (SBE) in rat mesangial cells, leading to the increase in connective tissue growth factor downstream of TGF-β signaling [40]. This was also seen by Wu et al. in hepatic progenitor cells [38]. We also found an upregulation of connective tissue growth factor (CTGF) (2.95-fold upregulation, FDR p-value = 0.0124, Supplemental Table 1), suggesting rapamycin induces the TGF-β/Smad/CTGF pathway in PMSCs as well. From the PPI analysis, the CXCL8 (IL-8) gene has interactions with IGF1R and TGFBR1 shown by the focused interaction network (Fig. 4B), meaning its upregulation is likely due to alterations in IGF-1 and TGF-β signaling pathways. TGF-β and IGF-1 signaling have been linked to both upregulation and downregulated IL-8 [41–44], thus, it is still unclear which alteration is leading to its upregulation. However, most evidence leans toward IL-8’s upregulation being dependent on the TGF-β pathway, evidenced by some studies showing increased IL-8 secretion after TGF-β1 treatment [44]. The PI3K/Akt/Snail axis has been linked to IL-8 transcription and secretion, which then positively regulates its own receptor, C-X-C chemokine receptor 1 and 2 (CXCR1/2), which could explain the continuous rise in IL-8 secretion of rapamycin-treated PMSC sheets (Fig. 3A) [45, 46]. TGF-β has also been shown to activate the PI3K/Akt pathway [47–49]. Therefore, it is possible that IL-8 is upregulated via a TGF-β/PI3K/Akt/Snail axis. However, this needs to be explored further.

## Conclusion

When grown in high density monolayer (sheet) culture, PMSCs have great potential for bioengineering applications, including bone tissue repair; however, the volume and culture conditions required to produce PMSC sheets induces senescence. In this report, we show for the first time to our knowledge that rapamycin can prolong the use of PMSCs in high density monolayers, decreasing markers of senescence, decreasing apoptosis, and modulating inflammatory cytokines. We found rapamycin upregulated IL-8 secretion in long term sheet culture. To further support the significance of IL-8 in these cells, Li et al. found that IL-8 is important for PMSC survival and proliferation, and when knocked out, PMSCs undergo accelerated senescence [50]. We further explored the molecular causes of IL-8 secretion with a microarray gene expression analysis, which indicated an increase in TGF-β/PI3K/Akt and TGF-β/Smad axis are likely involved. Along with TGFR1, TGFB2 was upregulated, which is a well characterized regulator of immune function (anti-inflammatory) and blood vessel formation, as well as key regulator of bone repair and maintenance [29, 51, 52].

With an interest in bone healing applications, the literature has conflicting findings on rapamycin’s effect on osteogenesis and osteoblast function [53–58]. Given that rapamycin resulted in a stark slowing of cellular metabolic processes, it is unlikely that rapamycin treated PMSCs themselves will more rapidly differentiate and contribute to the bone healing effect, as we found in our preliminary experiments (Supplemental Fig. S1B). However, it is more likely that the immunomodulatory effect will have the greatest influence on the bone healing process via transplantation. IL-8 is a potent chemoattractant of pro-healing immune cells, including in osteoprogenitors to the site of bone defects [31, 32, 59]. Further, Cen et al., found that the rapamycin-induced increase in IL-8 and TGF-β1 in MSCs leads to improved CD4 + T-cell migration and shifted to a predominantly regulatory T-cell (Treg) population vs the pro-inflammatory T helper 1 (Th1) subtype [60]. The current report is the first to our knowledge to show this finding in PMSC sheets, as well as suggest this finding is a result of mTOR-dependent differential feedback resulting in increased IGF-1/Akt and TGF-β pathways. Further, specific target genes identified in this study may lay the groundwork to explore future therapeutic targets and synergistic molecules for the continued improvement of not only the maintenance of MSCs in culture, but their therapeutic potential.

### Supplementary Information

Below is the link to the electronic supplementary material.

ESM 1 (XLSX 43.2 KB)

ESM 2 (DOCX 512 KB)

ESM 3 (JPG 248 KB)

## Data Availability

All data are available upon request to the corresponding author.
