# Supplementary material for: Rapamycin Inhibits Senescence and Improves Immunomodulatory Function of Mesenchymal Stem Cells Through IL-8 and TGF-β Signaling
Source: Stem Cell Rev Rep. 2024 Feb 10;20(3):816–26. doi: 10.1007/s12015-024-10682-x (PMC10984889; doi:10.1007/s12015-024-10682-x)
Supplement: Supplementary file 2 — (DOCX 512 KB) [file 12015_2024_10682_MOESM2_ESM.docx]

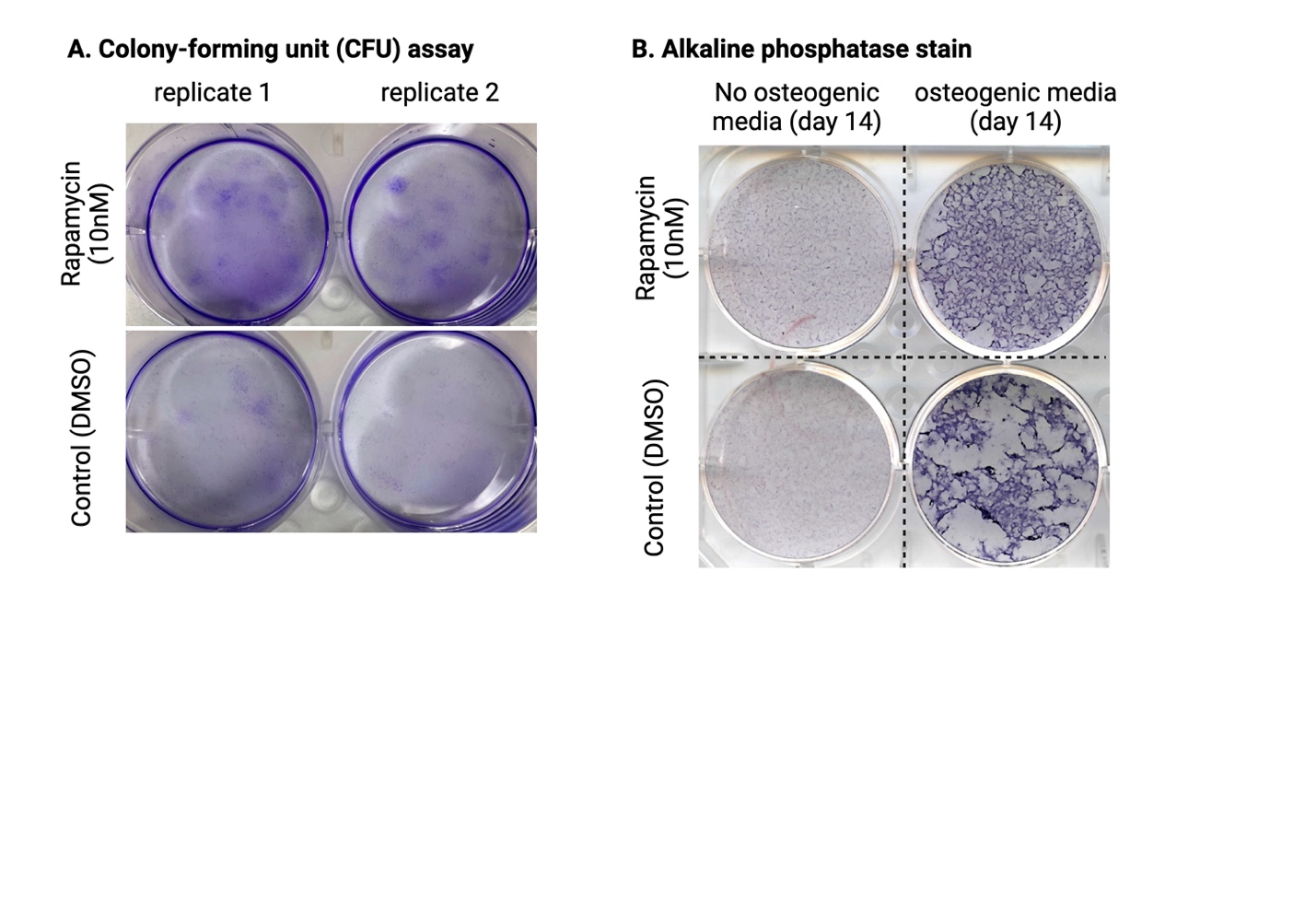


**Figure S1**: Colony Forming Unit (CFU) and Osteogenic Differentiation Preliminary Experiments. (**A**) After 4 days of high density PMSC sheet culture and treatment with either 10nM rapamycin or vehicle control (DMSO), the PMSCs were detached and seeded for CFU assay at 100 cells per cm^3^. The rapamycin treated PMSCs had retained viability as evident by the increased number of CFUs compared to the control. This increase in CFUs reflects the increase in the number of viable cells after sheet culture. (**B**) To investigate the osteogenic differentiation potential PMSC sheets, the PMSCs were first cultured for 4 days (high density sheet culture) with either 10nM rapamycin or vehicle DMSO. On day 5, the treatment media was exchanged for osteogenic media (MesenCult^TM^ Osteogenic Differentiation Kit, STEM CELL Technologies) and cultured for 14 days with 48hr media changes. Beginning around day 7 of osteogenic culture, the control plate began to show areas of cell detachment and the formation of niduses with intense ALP staining, which is shown most prominently at day 14 (**B, bottom right image**). This phenomenon is likely due to many cells undergoing apoptosis and necrosis, leading to calcific niduses of osteogenic activity and cell debris. However, the rapamycin treated PMSCs retained osteogenic potential (while less than the control) and had improved culture plate adhesion (**B, top right image**), likely due to less apoptosis/necrosis and the improved capacity for the treated cells to withstand cellular stress. Considering the decrease in osteogenic ability, but seemingly improved cellular resilience, this finding led to the further investigation of rapamycin’s role in immunomodulation and cellular function on the functional and molecular level.


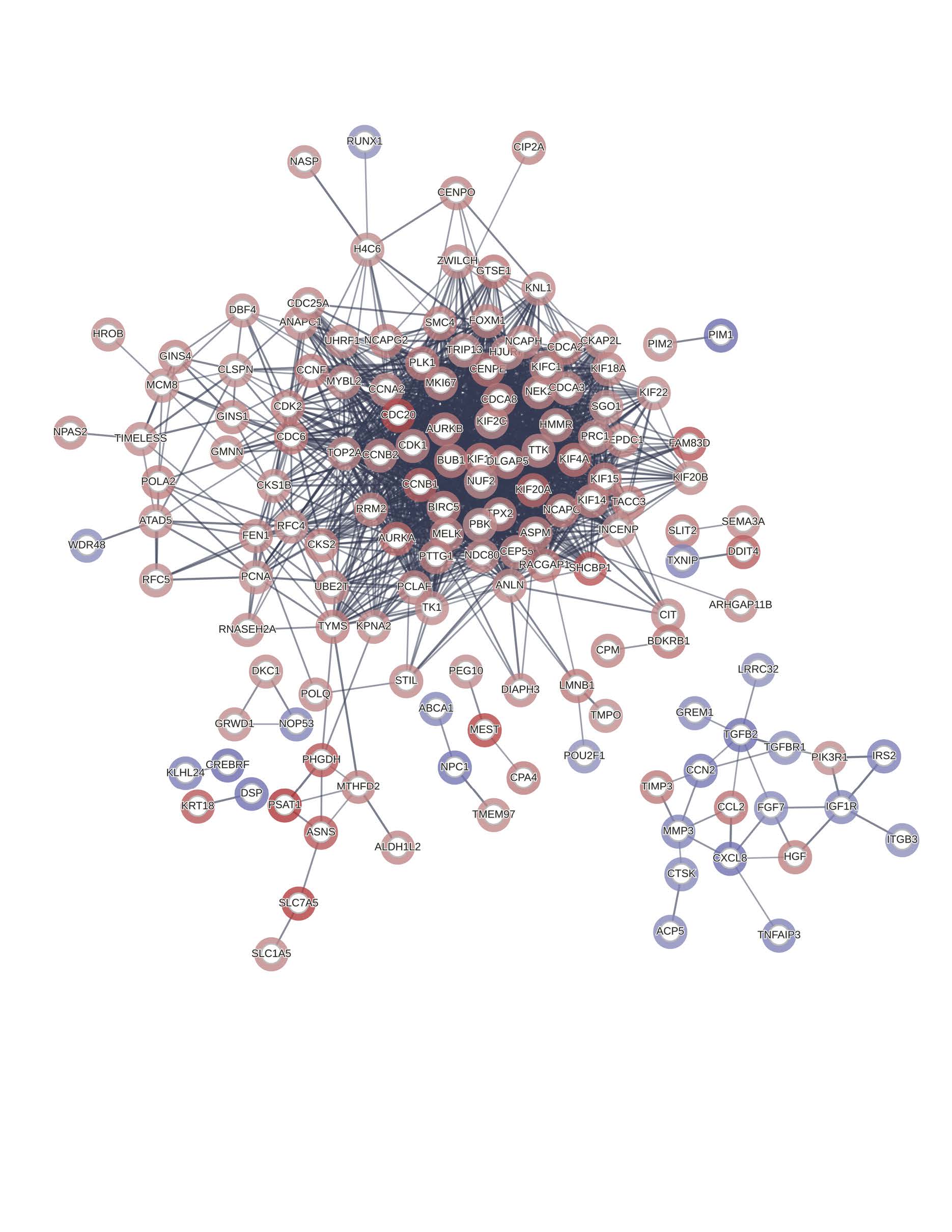


**Figure S2**: *Full Protein-Protein Interaction (PPI) n=Network*. Individual genes with a significant 2-fold difference in gene expression (and false discovery rate-adjusted p-value<0.05) were selected for input into the search tool for retrieval of interacting genes (STRING) (https://string-db.org) database using the geneset-based analysis. The interaction sources consisted of STRING’s text mining, database, experiments, co-occurrence, and co-expression with an interaction score of greater than 0.7 (high confidence) to construct the PPI network. Nodes represent proteins encoded by their labelled gene locus. Halo color and intensity signifies gene down-regulation (red) or up-regulation (blue) and fold-change value, respectively. The nodes correspond to the gene/proteins and the edges represent the interactions. After network analysis and removing unconnected nodes, 310 nodes were identified
