# Supplementary figures and images for: Rapamycin Inhibits Senescence and Improves Immunomodulatory Function of Mesenchymal Stem Cells Through IL-8 and TGF-β Signaling
Source: Stem Cell Rev Rep. 2024 Feb 10;20(3):816–26. doi: 10.1007/s12015-024-10682-x (PMC10984889; doi:10.1007/s12015-024-10682-x)

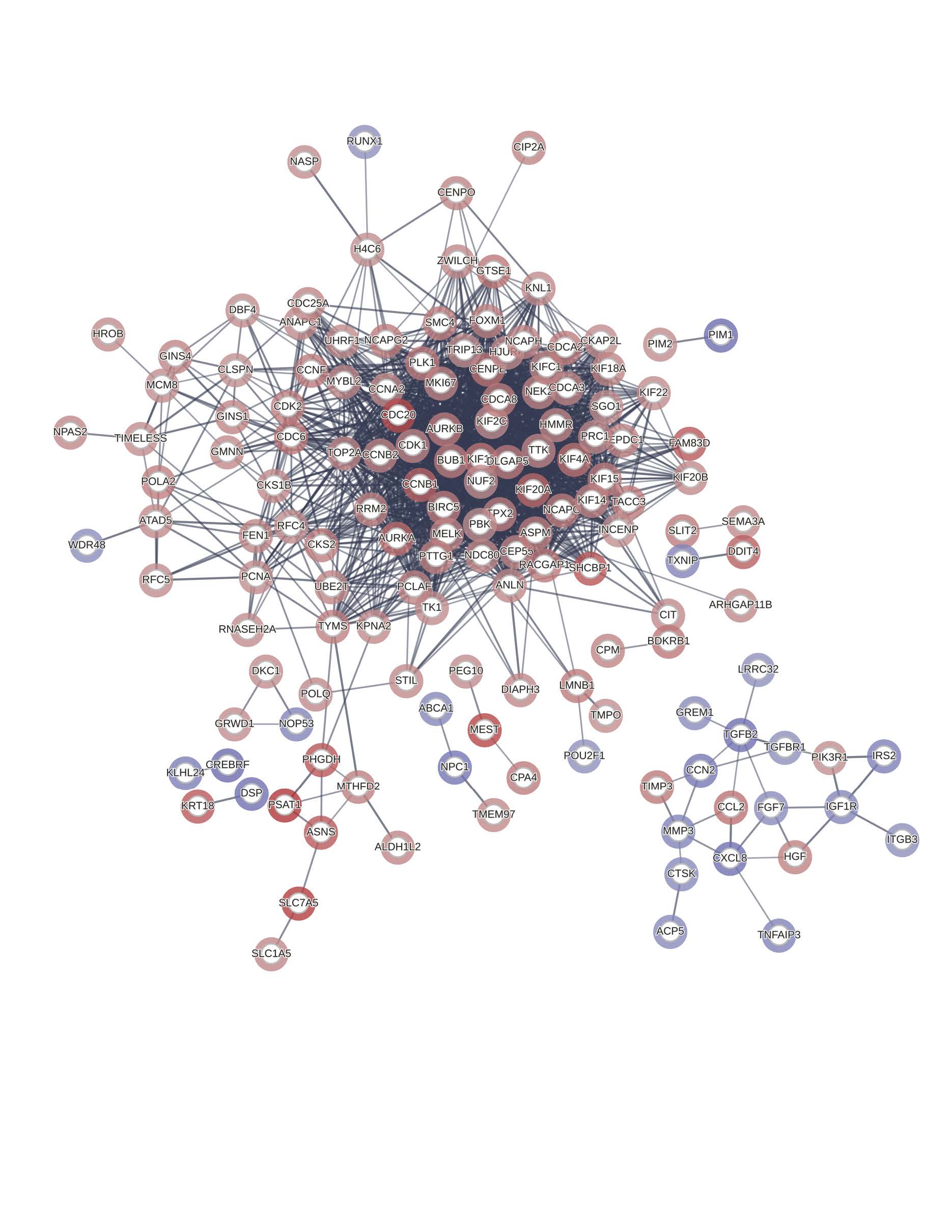

Supplement: Supplementary file 3 — (JPG 248 KB) [file 12015_2024_10682_MOESM3_ESM.jpg]
